# A Method for Identifying Mouse Pancreatic Ducts

**DOI:** 10.1089/ten.tec.2018.0127

**Published:** 2018-08-01

**Authors:** Chiemi Nakajima, Kenji Kamimoto, Katsuhiro Miyajima, Masahito Matsumoto, Yasushi Okazaki, Kazuo Kobayashi-Hattori, Makoto Shimizu, Takumi Yamane, Yuichi Oishi, Ken Iwatsuki

**Affiliations:** ^1^ Department of Nutritional Science and Food Safety, Faculty of Applied Bioscience, Tokyo University of Agriculture, Tokyo, Japan.; ^2^ Department of Developmental Biology, Washington University School of Medicine in St. Louis, St. Louis, Missouri.; ^3^ Department of Advanced Diabetic Therapeutics and Metabolic Endocrinology, Juntendo University, Tokyo, Japan.; ^4^ Diagnostics and Therapeutics of Intractable Diseases, Intractable Disease Center, Juntendo University, Tokyo, Japan.; ^5^ Department of Nutritional Science, Faculty of Applied Bioscience, Tokyo University of Agriculture, Tokyo, Japan.

**Keywords:** partial duct ligation, stem cells, differentiation, Ngn3

## Abstract

Proper identification of pancreatic ducts is a major challenge for researchers performing partial duct ligation (PDL), because pancreatic ducts, which are covered with acinar cells, are translucent and thin. Although damage to pancreatic ducts may activate quiescent ductal stem cells, which may allow further investigation into ductal stem cells for therapeutic use, there is a lack of effective techniques to visualize pancreatic ducts. In this study, we report a new method for identifying pancreatic ducts. First, we aimed to visualize pancreatic ducts using black, waterproof fountain pen ink. We injected the ink into pancreatic ducts through the bile duct. The flow of ink was observed in pancreatic ducts, revealing their precise architecture. Next, to visualize pancreatic ducts in live animals, we injected fluorescein-labeled bile acid, cholyl-lysyl-fluorescein into the mouse tail vein. The fluorescent probe clearly marked not only the bile duct but also pancreatic ducts when observed with a fluorescent microscope. To confirm whether the pancreatic duct labeling was successful, we performed PDL on Neurogenin3 (Ngn3)-GFP transgenic mice. As a result, acinar tissue is lost. PDL tail pancreas becomes translucent almost completely devoid of acinar cells. Furthermore, strong activation of Ngn3 expression was observed in the ligated part of the adult mouse pancreas at 7 days after PDL.

## Impact Statement

Although the result of partial duct ligation (PDL) provides us with an excellent model for pancreas regeneration after the injury, the technique involves ligation of a small portion of the tissue so that the results of PDL often varies experiment to experiment, leading researchers inconsistent conclusions. The main reason for this is that pancreatic ducts are thin and translucent. In addition, there are chances to ligate blood vessels together with pancreatic ducts that make the result more complicated. We therefore tried to find a way of labeling pancreatic ducts and finally found a simple method to fluorescently label them in live animals. Using this method, we have increased the success rate of PDL and therefore could obtain reproducible results in regeneration studies.

## Introduction

More than 420 million people suffer from diabetes worldwide.^1^ Slowing the increasing prevalence of diabetes is a major challenge in the medical field, because no effective drug presently exists. Transplantation of pancreatic islets is a reliable diabetes treatment that is presently available.^2,3^ Islets can be generated from pancreatic progenitor cells that emerge when the tissue is damaged. However, where these progenitor cells reside remains controversial.^4–8^ It is generally thought that beta cell growth occurs only by self-renewal of mature beta cells under normal physiological conditions.^9–11^ In other studies, it has been reported that beta cell progenitors can be generated by partial duct ligation (PDL) in adult mice.^12–14^ The procedure involves ligation of the pancreatic duct that drains pancreatic enzymes into the duodenum. This ligation model could be a valid analytical tool to study the mechanism of pancreatic beta cell neogenesis. However, the outcomes of studies using the ligation technique appear to vary across laboratories; some groups show that “the response to PDL induces not only measurable but also large increases in beta cell mass,”^6,12–17^ whereas the other group observed that “there is no solid evidence that new beta cells occurred in PDL pancreas.”^18–20^ Although De Groef *et al.* established the PDL surgical procedure,^21^ the success rate of ligation varied from experiment to experiment. The main reason for this is that the pancreatic duct is thin and often difficult to identify. To overcome this problem, methods to precisely identify and ligate pancreatic ducts are necessary. Furthermore, visualizing pancreatic ducts greatly enhances our ability to study the role of pancreatic ducts in numerous aspects of pancreatic growth, development, and function. To identify pancreatic ducts, Guo *et al.* described ductal labeling methods using a recombinant adeno-associated virus with a duct-specific Sox9 promoter infused into mouse pancreatic ducts.^22^ However, there is no report for a simplified method to identify pancreatic ducts in native tissues. In this study, we report a new, simple labeling technique to reveal the architecture of mouse pancreatic ducts. Using this method, we generated a PDL model and observed the same results as previous reports such as loss of acinar cells and ductal neogenesis.^13,14,21^ Moreover, upregulation of Ngn3 (neurogenin3), a well-established endocrine cell marker^23^ and a well-established marker for embryonic islet cell progenitors,^23–25^ was observed in the ligated pancreas at 7 days after PDL.

## Materials and Methods

### Animals

All animals used in this study were obtained, housed, cared for, and used in accordance with the “Guiding Principles in the Care and Use of Animals” published by the Animal Care Committee of Tokyo University of Agriculture (Ethics identification number: 140014). Wild-type C57BL/6J mice and Jcl: ICR mice were purchased from CLEA Japan (Tokyo, Japan). The Ngn3-GFP mice were backcrossed (for eight generations) using a C57BL/6 background. The Ngn3-GFP mouse has been described previously.^26^ Animals were maintained in a 12 h light cycle providing food and water *ad libitum*.

### Visualizing mouse pancreatic ducts using black ink

**FIG. 1.**
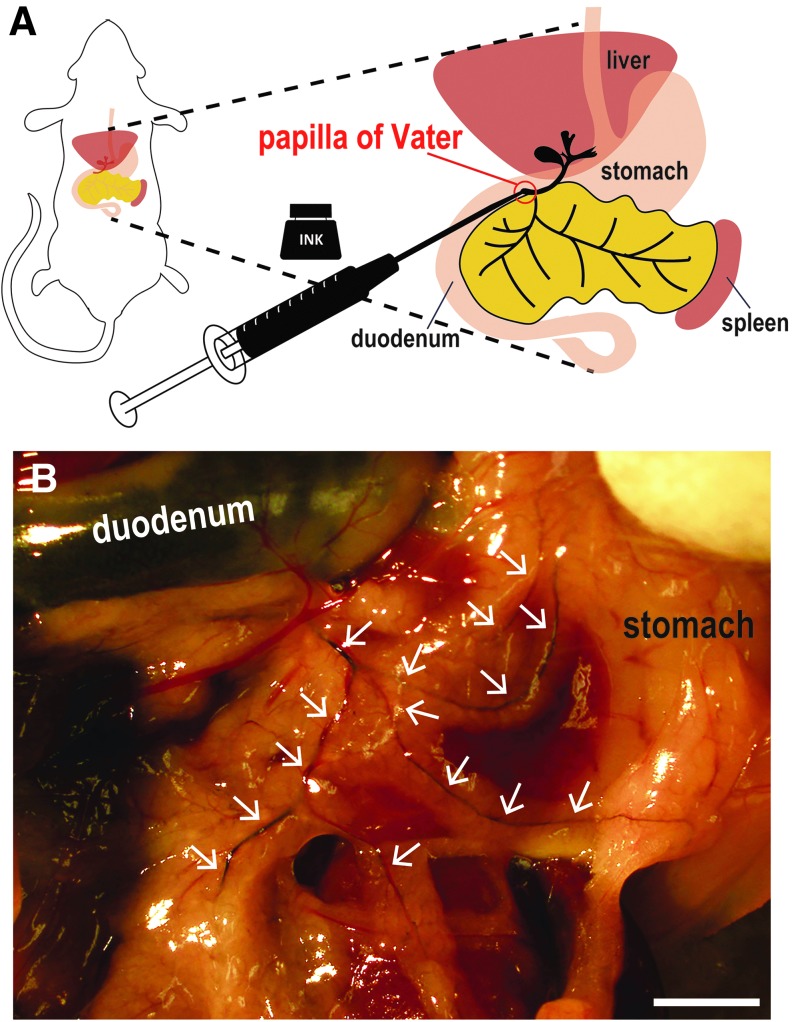
Visualization of pancreatic ducts using black ink. **(A)** Schematic figure of the mouse gut and site of ink injection. Black waterproof fountain pen ink (SPC-200) was injected into the pancreatic ducts through the bile duct. **(B)** Pancreatic ducts (*white arrows*) became visible after black ink injection. Scale bar, 2 mm.

Sodium pentobarbital was purchased from Kyoritsu Seiyaku Corporation (Tokyo, Japan). SPC-200, black, waterproof, fountain pen ink was purchased from Platinum Japan Corporation (Tokyo, Japan). Microhematocrit capillary tubes (22-362-566) were purchased from Fisher Scientific Corporation (Tokyo, Japan). Fine glass capillaries were made by stretching heated microhematocrit tubes. Then, a 1 mL syringe was connected to a glass capillary through a silicon tube. Adult mice were sacrificed by excessive anesthesia (sodium pentobarbital anesthesia at a dose of 100 mg/kg body weight) and subjected to laparotomy. The spleen, the stomach, and the duodenum were removed using sterile tweezers or swab, according to the method of De Groef *et al.*^21^ Pancreatic ducts branch from the bile duct, and the bile duct flows into the duodenum. We transferred the intestinal wall with a microhematocrit capillary tube slowly to locate the papilla of Vater, then injected 10–15 μL of undiluted SPC-200 ink, slowly into the bile duct retrogradely, as described in recent studies.^27^ The ink was infused onto the bile duct and the pancreatic duct through the papilla of Vater (Fig. 1A).

### Labeling of pancreatic ducts using a fluorescent reagent and PDL

**FIG. 2.**
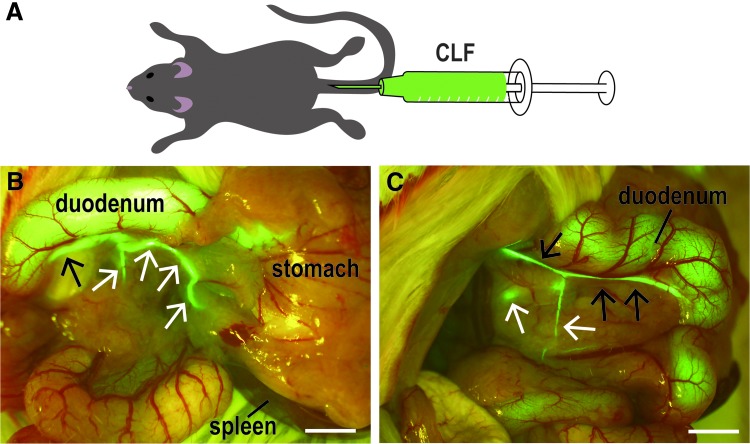
Visualizing pancreatic ducts after fluorescent probe injection. **(A)** Mice were injected with CLF (100 μg/mL) at a dose of 350 μg/kg body weight by tail vein injection. **(B,C)** CLF clearly marked not only the bile duct (*black arrows*) but also pancreatic ducts (*white arrows*) under the fluorescent microscope. C is an image of the underside of B. Scale bar, 2 mm. CLF, cholyl-lysyl-fluorescein.

Chlorhexidine gluconate was purchased from Sumitomo Dainippon Pharma (Osaka, Japan). Pentobarbital sodium was purchased from Kyoritsu Seiyaku Corporation. Cholyl-lysyl-fluorescein (CLF) was purchased from CORNING (Woburn). CLF is a fluorescent bile salt whose biological behavior closely resembles that of naturally occurring cholylglycine and is a potential agent for assessing liver function *in vivo*.^28^ CLF fluorescently labels the bile duct. According to the previous report, intestinal uptake of CLF was negligible when compared with taurocholate in mice, because absorbed CLF is transported back into the blood stream by the ABCC3 transporter.^29^ Enterohepatic circulation of CLF is reabsorbed into the portal vein and then discharged. All dissecting instruments were sterilized by 0.5% chlorhexidine gluconate to minimize infection. A heating pad was provided at of 38°C for temperature stabilization during surgery. The animals (Jcl: ICR mice) were dissected under sodium pentobarbital anesthesia at a dose of 30 mg/kg body weight and put into an adjuster. Then, animals were injected with CLF (100 μg/mL) at a dose of 350 μg/kg body weight by tail vein injection (Fig. 2A). After 10 min, a laparotomy was performed through a midline abdominal incision. The spleen, the stomach, and the duodenum were removed as described above. The pancreatic duct visualized by the fluorescent substance using a stereoscopic microscope was ligated at the tail region using a sterilized thread. Ligation was performed to minimize damage to the underlying blood vessels, such as the superior pancreaticoduodenal artery, the inferior pancreaticoduodenal artery, and the pancreatic part of the splenic artery. The incision was sewn up, and the mouse was placed in the warm recovery area. At 1, 3, and 14 days after CLF administration (*n* = 3, respectively), the pancreas, liver, duodenum, kidney, and spleen were fixed with 4% paraformaldehyde (PFA), and the tissues were paraffin-embedded using standard techniques, and thin sections were made (3–5 μm). The sections were stained with hematoxylin and eosin (HE). HE-stained samples were examined histopathologically. At 7 days after CLF administration, total protein, albumin, alanine aminotransferase, total cholesterol, triglyceride, bile acid, and total bilirubin in sera were biochemically measured by Oriental Yeast (Tokyo, Japan).

### Immunohistochemistry

Antibodies used were rat anti-mouse CD326 (BD Bioscience, Tokyo, Japan), rabbit antiamylase (ab21156), goat anti-PDX1 (ab47383; Abcam, Cambridge, United Kingdom), and goat anti-GFP (ROCKLAND, Limerick, Ireland). Rabbit anti-CK19 antibodies were a gift from Prof. Atsushi Miyajima.^27,30^ Seven days after PDL administration, Ngn3-GFP mice (*n* = 3) were infused with 10 mL phosphate-buffered saline (PBS) followed by 10 mL 4% PFA in PBS, using a peristaltic pump (ATTO, Tokyo, Japan). The pancreatic tissues were then harvested and fixed for 2 h at 4°C in 4% PFA in PBS, transferred to 30% sucrose in PBS, and stored at 4°C overnight. Tissues were dissected and embedded in O.C.T compound (Sakura Finetek Japan). Then, 12 μm frozen sections were prepared and incubated in 0.3% Triton X-100 and 2% donkey serum for 1 h at room temperature. The primary antibodies were added to the section in a humidified chamber and incubated overnight at 4°C. Sections were washed in PBS, incubated with the secondary antibodies (Alexa Fluor 555 or 594 IgG against primary antibodies from Invitrogen, Tokyo, Japan) for 1 h, and rinsed in PBS. For counterstaining, nuclei were labeled with DAPI (Nacalai Tesque, Kyoto, Japan).

## Results

### Visualization of mouse pancreatic ducts using black ink

By injecting black ink into the bile duct through the duodenum and the papilla of Vater (Fig. 1A), both the bile duct and pancreatic ducts of the head and tail region were clearly labeled (Fig. 1B). As translucent pancreatic ducts became obvious, the main pancreatic ducts as well as fine pancreatic ducts could be identified (white arrowheads in Fig. 1B). Therefore, by ink injection, the precise architecture of the mouse pancreatic ducts was revealed.

### Visualization of pancreatic ducts using a fluorescent probe

Although the ink injection strategy allowed us to identify pancreatic ducts, we next screened for chemicals that are not harmful to the animal, so that it could be used in live animals. We found the candidate compound CLF, which is a fluorescently labeled analog of bile acid. After administration of CLF through the tail vein, we observed that this fluorescent probe labeled the bile duct. Furthermore, CLF also labeled pancreatic ducts (Fig. 2B, C). Using this method, both head and tail pancreatic ducts were clearly visualized in live animals. To fully understand this method for visualizing pancreatic ducts, we investigated whether this method affects any other organs and tissues. The results of the histopathological observation revealed that there were no abnormalities in any other tissues in all animals, including 1, 3, and 14 days after the operation (*n* = 3; Supplementary Fig. S1; Supplementary Data are available online at www.liebertpub.com/tec). Biochemical assay using sera showed no significant damage of tissues by CLF administration (Supplementary Table S1).

### PDL operation after CLF injection

To examine if the use of our new CLF duct visualization method increases success rates of PDL, we performed PDL on Ngn3-GFP reporter mice. The transgenic mice express a GFP reporter gene under the control of a Ngn3 promoter. We detected GFP signal in enteroendocrine cells of the duodenum, jejunum, and stomach where Ngn3 expression has been reported^31^ (Supplementary Fig. S2A–C). In normal condition, GFP signal could not be detected in the pancreas of Ngn3-GFP mice (Supplementary Fig. S2D). After PDL operation, GFP reporter signals were observed around outside of the ductal lining.

**FIG. 3.**
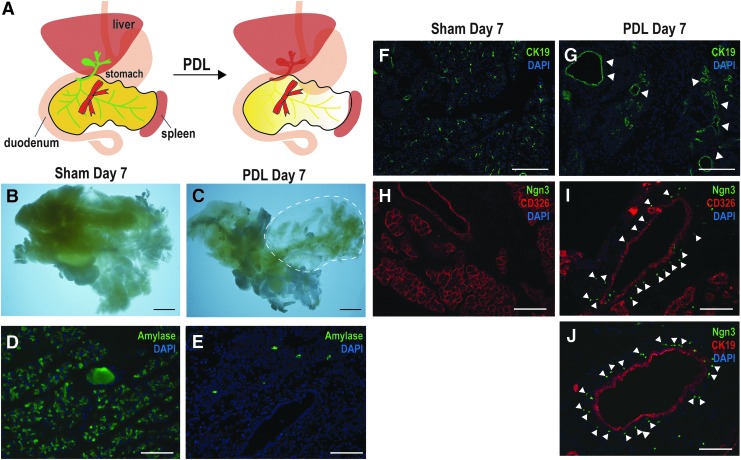
Partial duct ligation using the CLF visualizing method. **(A)** A schematic representation of the ligation site and the result of PDL. The pancreatic duct labeled by CLF was ligated at the tail region. **(B, C)** Representative photos of the pancreas 7 days after PDL (*n* = 3). Acinar cells of the tail region became translucent as a result of autolysis after ligation (**C**, *dotted circle*). Scale bar, 2 mm. **(D–J)** Immunohistochemical analysis of the PDL-treated pancreas with sham-operated tissue serving as a control. **(D, E)** Amylase immunoreactivity was observed from the sham-operated tail region of the pancreas **(D)**, while most of amylase positive cells were gone at Day 7 after PDL operation **(E)**. **(F, G)** Upregulation of CK19 expression (*white arrowheads*) was observed in the PDL tissue section, indicating ductal neogenesis. **(H, I)** CD326, a general epithelial marker was detected in both ductal and acinar cells of sham tissue, but could be detected only from ductal cells after PDL operation. **(I, J)** A strong but specific expression of Ngn3-GFP (*white arrowheads*) was observed peripheral to the pancreatic ducts of the PDL pancreas. Scale bars: **D, E, H–J**, 100 μm and **F, G**, 250 μm. Ngn3, neurogenin3; PDL, partial duct ligation.

The pancreatic duct draining the exocrine enzymes from the tail of the pancreas was ligated while the organ's head, located adjacent to the stomach and duodenum, remained unaffected. The ligature is applied downstream of the splenic part or tail of the pancreas, referred to as the “PDL tail.” As has been previously reported,^21^ PDL seemed to induce inflammation and acinar cell atrophy resulting a gradual loss of acinar tissue. In contrast, acinar cell death does not occur in the “head” region of the PDL pancreas. Age-matched male or female mice underwent sham surgery, recapitulating all steps of PDL surgery, except the ligation of the pancreatic duct. Pancreatic tissues were harvested 7 days postsurgery. Using this new visualizing method, almost all PDLs were performed correctly. Success rate of the PDL with a new method was 88% (22 successes out of 25 trials) while 10% (1 success out of 10 trials) with a conventional method. When the tail pancreatic PDL was performed, acinar cells were depleted, making the PDL tail pancreas translucent (Fig. 3B, C). Depletion of acinar cells was also confirmed by detecting amylase, an acinar cell marker (Fig. 3D, E, Supplementary Fig. S5), and by hematoxylin-eosin staining (data not shown). Furthermore, ductal reconstruction was observed in the PDL section as detected by CK19 antibodies (Fig. 3F, G). Interestingly, Ngn3 expression was upregulated during pancreatic regeneration followed by PDL. A strong Ngn3 signal represented by GFP expression was observed outside lining of the CD326 and CK19 positive ductal markers 7 days postsurgery (Fig. 3I, J, Supplementary Fig. S3). We failed to detect any GFP signal in sham-operated pancreas of Ngn3-GFP mice (Fig. 3H). We have verified that green fluorescent signals from Ngn3-GFP mice are derived from GFP using anti-GFP antibodies (Supplementary Fig. S4, top panels). These data suggest that Ngn3^+^ cells originate from pancreatic ductal cells.

## Discussion

In this study, we demonstrated a new, simple imaging technique to reveal the architecture of mouse pancreatic ducts using both black waterproof fountain pen ink and the fluorescein-labeled bile acid analog CLF. Both reagents clearly labeled pancreatic ducts as well as the bile duct and allowed us to track major ducts. By using CLF as an artificial bile acid, we succeeded in labeling pancreatic ducts in real time, low invasively. Accordingly, the visualized pancreatic duct could be ligated at the intended position, and PDL was pathologically and histologically confirmed after 1 and 2 weeks. Thus, we have established a new technique for PDL using this simple imaging technique. As described earlier, there are contradictory results over the exact origin of these progenitor cells.^4,32^ There are at least three models to explain regenerative activities of islets. The first model shows that adult pancreatic cells produce progenitor cells upon injury. In this model, pancreatic duct cells obtain ability to differentiate into both endocrine and exocrine cells.^5,6^ The second model suggests self-duplication of adult pancreatic beta-cells.^7^ The third model utilizes acinar cells to obtain transdifferentiation activities.^8^ These discrepancies could be attributed to a combination of factors such as bodyweight, sex, and age of the mice, but, most importantly, differences in surgical technique.^11,21^ Since pancreatic ducts cannot easily be identified, ligating the pancreatic duct may result in ligation of some blood vessels as well. These surgical complications may be the source of outcome variation. Because our new method does not require any special techniques and is highly reproducible, we believe our method could overcome these previous complications.

In the present study, we utilized Ngn3-GFP reporter mice to determine whether GFP-marked pancreas progenitor cells appear after PDL. It has been reported that Ngn3 expression is detected in enteroendocrine progenitor cells in the stomach and small intestines.^31^ After PDL was performed, our samples became devoid of acinar cells and ductal neogenesis could be observed, accompanied by upregulation of Ngn3 expression proximal to pancreatic ducts. Our results are consistent with the previous work by Xu *et al.*, suggesting that Ngn3^+^ cells originate from pancreatic ductal cells.^13^ Other transcription factor reporter mice, such as Nkx6.1^33^ and MafA,^34^ could serve as beneficial tools in investigating pancreatic cell differentiation and determining the origin of pancreatic progenitor cells after lesioning of the pancreas.

In conclusion, the newly established method for imaging the pancreatic duct has potential to provide an excellent model for pancreatic regeneration and differentiation, to assist for the fundamental understanding of the mechanism of pancreatic stem/progenitor cells to benefit future therapies and drug screening.

## Supplementary Material

Supplemental data

Supplemental data

Supplemental data

Supplemental data

Supplemental data

Supplemental data
