# Supplementary material for: A Method for Identifying Mouse Pancreatic Ducts
Source: Tissue Eng Part C Methods. 2018 Aug 1;24(8):480–5. doi: 10.1089/ten.tec.2018.0127 (PMC6088256; doi:10.1089/ten.tec.2018.0127)
Supplement: Supplemental data [file Supp_Fig1.pdf]

Supplementary Data

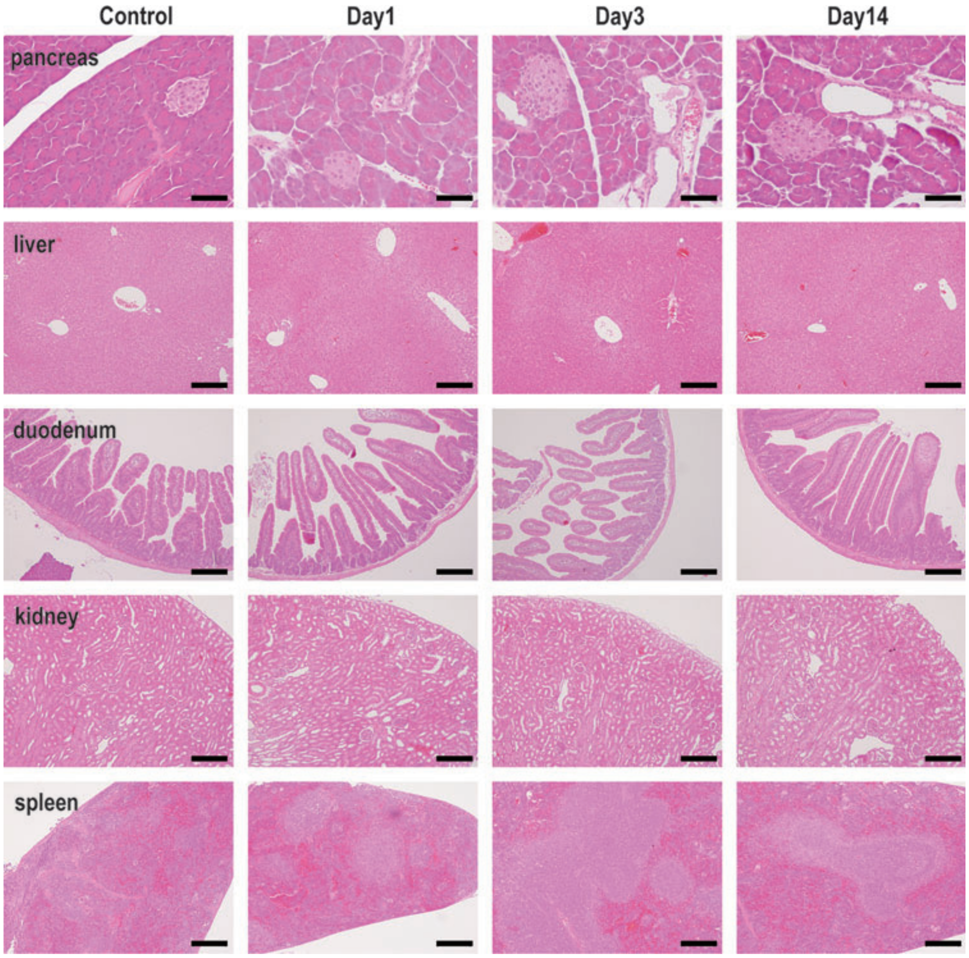

SUPPLEMENTARY FIG. S1. CLF administration did not cause any additional tissue damage. At 1, 3, and 14 days after CLF injection ($n = 3$, respectively), tissues (pancreas, liver, duodenum, kidney, and spleen) were fixed, sectioned, and HE stained. No abnormalities were found in any of the observed tissues. Scale bar, 500 μm. CLF, cholyl-lysyl-fluorescein; HE, hematoxylin and eosin.
