# Supplementary material for: A Method for Identifying Mouse Pancreatic Ducts
Source: Tissue Eng Part C Methods. 2018 Aug 1;24(8):480–5. doi: 10.1089/ten.tec.2018.0127 (PMC6088256; doi:10.1089/ten.tec.2018.0127)
Supplement: Supplemental data [file Supp_Table1.pdf]

Supplementary Table S1. Results of Biochemical Analysis Using Sera of Cholyl-Lysyl-Fluorescein-Treated Mice

	TP (g/dL)	ALB (g/dL)	ALT (IU/L)	T-CHO (mg/dL)	TG (mg/dL)	T-BIL (mg/dL)	TBA (μ mol/L)
Cont-1	4.4	3.0	68	80	40	0.02	4
Cont-2	4.2	3.4	52	62	54	0.06	6
Cont-3	4.2	3.3	102	66	51	0.06	6
CLF Day7-1	4.6	3.2	32	62	46	0.04	2
CLF Day7-2	4.2	3.0	40	68	46	0.04	6
CLF Day7-3	4.4	3.0	26	76	70	0.04	4

Seven days after CLF administration, sera were collected and analyzed for TP, ALB, ALT, T-CHO, TG, T-BIL, and TBA. Cont stands for control mouse without CLF administration.

ALB, albumin; ALT, alanine aminotransferase; CLF, cholyl-lysyl-fluorescein; TBA, total bilirubin; T-BIL, bile acid; T-CHO, total cholesterol; TG, triglyceride; TP, total protein.
