# Supplementary material for: A Method for Identifying Mouse Pancreatic Ducts
Source: Tissue Eng Part C Methods. 2018 Aug 1;24(8):480–5. doi: 10.1089/ten.tec.2018.0127 (PMC6088256; doi:10.1089/ten.tec.2018.0127)
Supplement: Supplemental data [file Supp_Fig2.pdf]

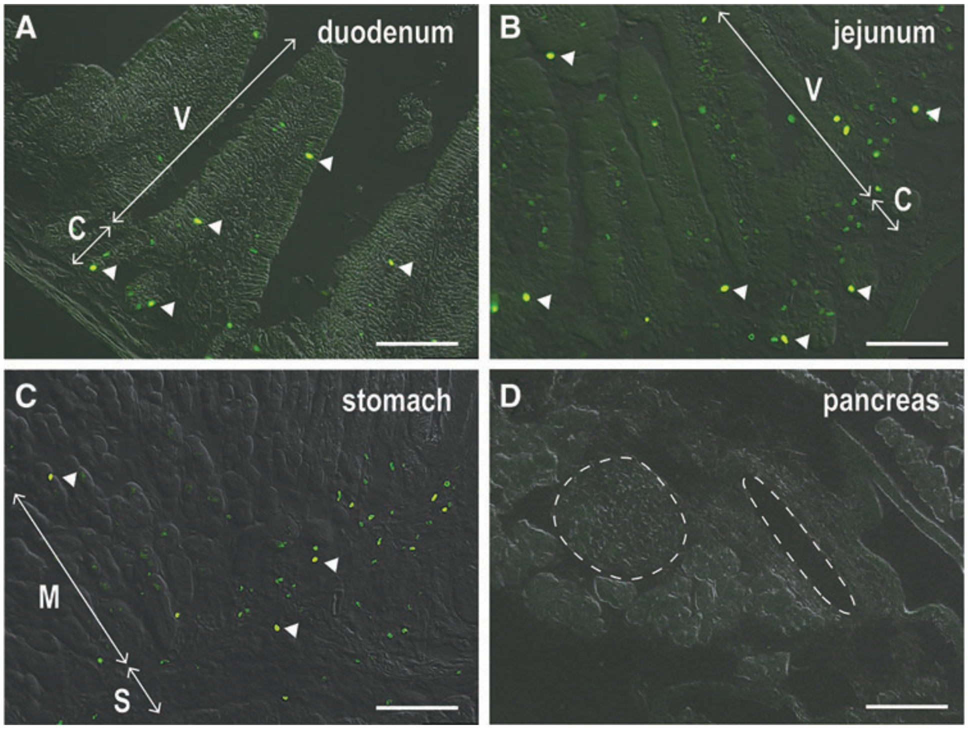

SUPPLEMENTARY FIG. S2. Representative images of sections from the Ngn3-GFP reporter mouse. Tissues from Ngn3-GFP mice were dissected, sectioned, and observed under a fluorescent microscope. **(A–C)** GFP signals were observed in the duodenum, jejunum, and stomach (*white arrowheads*); however, GFP signals were not detected in the pancreas **(D)** *Dotted lines* in D outline a pancreatic islet (*left*) and the pancreatic duct (*right*). Scale bars, 100 µm. C, crypt; M, mucosa; Ngn3, neurogenin3; S, submucosa; V, villi.
