# Supplementary material for: A Method for Identifying Mouse Pancreatic Ducts
Source: Tissue Eng Part C Methods. 2018 Aug 1;24(8):480–5. doi: 10.1089/ten.tec.2018.0127 (PMC6088256; doi:10.1089/ten.tec.2018.0127)
Supplement: Supplemental data [file Supp_Fig5.pdf]

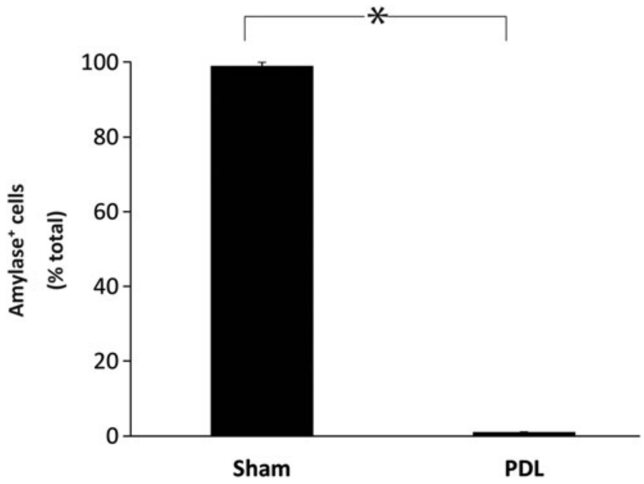

SUPPLEMENTARY FIG. S5. Quantitative analysis of acinar cells (amylase$^+$ cells) at 7 days after PDL. The number of amylase$^+$ cells in the pancreatic tissue sections was significantly reduced by PDL operation compared with sham operation (0.61% ± 0.47% in ligated tail vs. 98.6% ± 1.44% in sham tail. Data are mean ± SD, $n = 8$, $^*p < 0.05$). SD, standard deviation.
