# Supplementary material for: A Method for Identifying Mouse Pancreatic Ducts
Source: Tissue Eng Part C Methods. 2018 Aug 1;24(8):480–5. doi: 10.1089/ten.tec.2018.0127 (PMC6088256; doi:10.1089/ten.tec.2018.0127)
Supplement: Supplemental data [file Supp_Fig3.pdf]

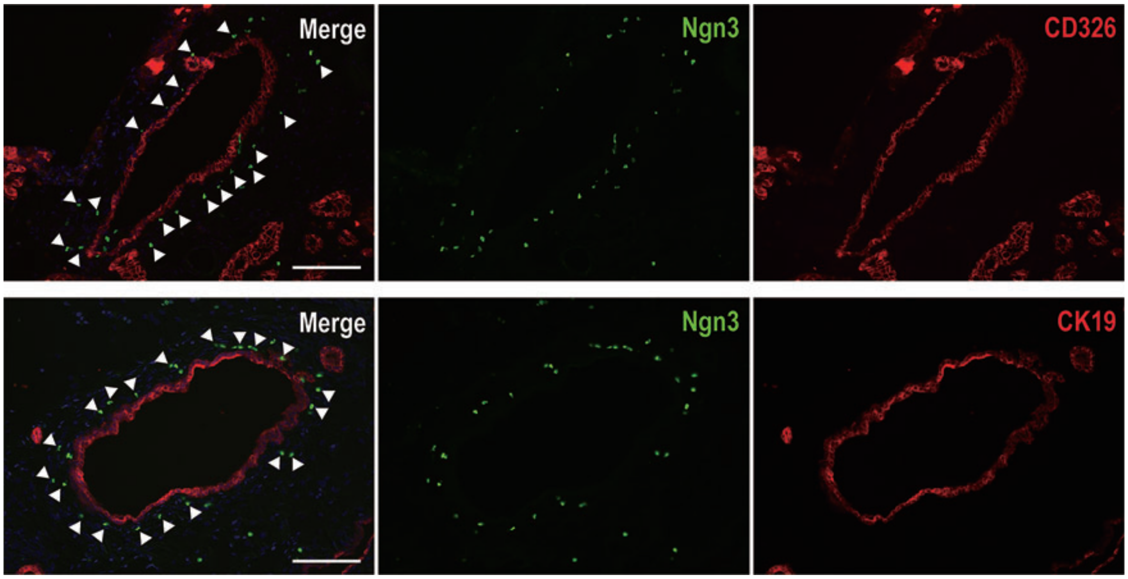

SUPPLEMENTARY FIG. S3. Merged images of Figure 3 (I, J) with their single fluorescent images. *Upper* and *lower panels* both show that Ngn3-GFP (*green*; *white arrowheads*) positive cells reside outside of the ductal linings that are labeled with antibodies against CD326 and CK19. Scale bars, 100 μm.
