# Supplementary material for: A Method for Identifying Mouse Pancreatic Ducts
Source: Tissue Eng Part C Methods. 2018 Aug 1;24(8):480–5. doi: 10.1089/ten.tec.2018.0127 (PMC6088256; doi:10.1089/ten.tec.2018.0127)
Supplement: Supplemental data [file Supp_Fig4.pdf]

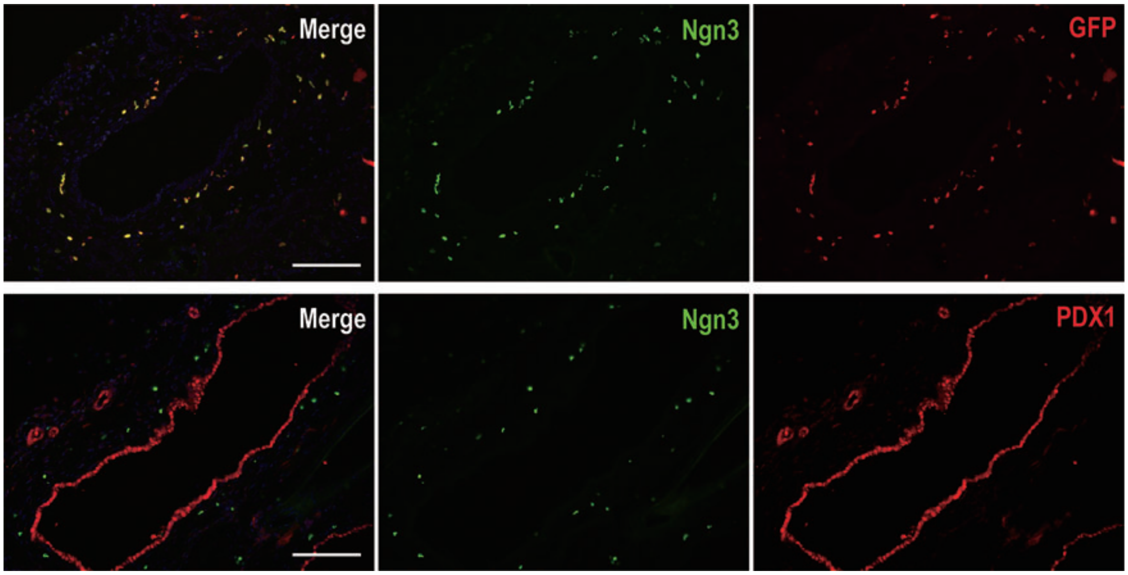

SUPPLEMENTARY FIG. S4. Antibody verification and PDX1 immunohistochemistry. Consecutive sections from PDL-operated Ngn3-GFP mice were subjected to immunohistochemistry using anti-GFP antibodies (*upper panel*) and anti-PDX1 antibodies (*lower panel*), respectively. Ngn3-GFP (*green*) signals were always marked with anti-GFP antibodies (*red*) suggesting that the antibody against GFP is reliable. PDX1, a marker for pancreatic progenitor cells, was found to be strictly localized to the pancreatic duct at 7 days after PDL treatment. Scale bars, 100 μm. PDL, partial duct ligation.
